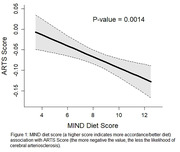# MIND diet, Cerebrovascular Health, and Cognition among community‐dwelling Older Adults

**DOI:** 10.1002/alz.086470

**Published:** 2025-01-09

**Authors:** Puja Agarwal, Melissa Lamar, Alifiya Kapasi, Arnold M Evia, Neelum T. Aggarwal, Laurel J Cherian, David A. Bennett, Sue E. Leurgans, Konstantions Arfanakis, Julie A. Schneider

**Affiliations:** ^1^ Rush Alzheimer’s Disease Center, Rush University Medical Center, Chicago, IL USA; ^2^ Rush Alzheimer’s Disease Center, Chicago, IL USA; ^3^ Rush University Medical Center, Chicago, IL USA; ^4^ Department of Neurological Sciences, Rush University Medical Center, Chicago, IL USA; ^5^ Rush University, Chicago, IL USA; ^6^ Rush Alzheimer's Disease Center, Rush University Medical Center, Chicago, IL USA

## Abstract

**Background:**

White matter hyperintensities (WMH) and cerebral arteriosclerosis involving thickening of the vessel wall and stenosis of brain arterioles are common in older adults and associated with poor cognition. The Mediterranean‐DASH Intervention for Neurodegenerative Disease (MIND) diet is associated with better cognition. Little is known about the association of the MIND diet with cerebrovascular outcomes and if this association mediates the link between diet and cognition. This study investigates the association of the MIND diet with WMH volume and arteriolosclerosis, and whether WMH and arteriosclerosis mediate the association between diet and cognition among community‐dwelling older adults.

**Method:**

The study includes 561 participants from an ongoing longitudinal cohort study, the Rush Memory and Aging Project with dietary and neuroimaging data available no more than a year apart. MIND diet scores were obtained using a valid >142‐item food frequency questionnaire. In‐vivo magnetic resonance imaging (MRI) data were collected using a 3T MRI scanner. WMH were segmented using a deep learning model. Arteriolosclerosis was assessed employing a fully automated in‐vivo MRI‐based marker named ARTS (ARTerioloSclerois). An ARTS score was generated for each participant (higher scores indicate a higher likelihood of arteriolosclerosis). Global cognition was assessed using 19‐battery tests. Linear regression models controlled for age, sex, MRI scanner, and education. Mediation analysis was done using structural equation modeling.

**Result:**

In our analytical sample (mean age (81 ± 7.5), 77% female, 16 ± 2.9 years of education a higher MIND diet score was associated with less WMH (β = ‐0.023, SE = 0.009, *p* = 0.015) and lower ARTS score (β = ‐0.013, SE = 0.004, *p* = 0.001). The effect estimates were stable when further controlled for BMI and physical activity. The MIND diet was associated with better global cognition (β = 0.0426, SE = 0.012, *p* = 0.0004), with an indirect association of 10.5% through ARTS score (β = 0.0045, SE = 0.0002, *p* = 0.038).

**Conclusion:**

Among community‐dwelling older adults a higher MIND diet score is associated with less WMH and a lower likelihood of small vessel arteriosclerosis. The association of the MIND diet with cognition is partially mediated by arteriosclerosis. Lifestyle approaches such as adherence to a specific diet may improve cognition by maintaining the cerebrovascular health of older adults.

**Fundings sources**: R01AG054476; R01AG17917; R01AG076143